# *Drosophila Ectoderm-expressed 4* modulates JAK/STAT pathway and protects flies against *Drosophila* C virus infection

**DOI:** 10.3389/fimmu.2023.1135625

**Published:** 2023-02-03

**Authors:** Zongliang Huang, Wei Wang, Pengpeng Xu, Shangyu Gong, Yingshan Hu, Yan Liu, Fang Su, Khalid Mahmood Anjum, Wu-Min Deng, Suping Yang, Jiyong Liu, Renjie Jiao, Jianming Chen

**Affiliations:** ^1^ Fujian Key Laboratory on Conservation and Sustainable Utilization of Marine Biodiversity, Fuzhou Institute of Oceanography, Minjiang University, Fuzhou, Fujian, China; ^2^ Sino-French Hoffmann Institute, School of Basic Sciences, Guangzhou Medical University, Guangzhou, Guangdong, China; ^3^ Department of Bioengineering and Biotechnology, College of Chemical Engineering, Huaqiao University, Xiamen, Fujian, China; ^4^ Department of Wildlife and Ecology, University of Veterinary and Animal Sciences, Lahore, Punjab, Pakistan; ^5^ Department of Biological Science, Florida State University, Tallahassee, FL, United States

**Keywords:** Ect4, Drosophila C virus, tyrosine phosphorylation, JAK/STAT pathway, innate immunity

## Abstract

Sterile alpha and HEAT/Armadillo motif-containing protein (SARM) is conserved in evolution and negatively regulates TRIF-dependent Toll signaling in mammals. The SARM protein from *Litopenaeus vannamei* and its *Drosophila* orthologue Ectoderm-expressed (Ect4) are also involved in immune defense against pathogen infection. However, the functional mechanism of the protective effect remains unclear. In this study, we show that Ect4 is essential for the viral load in flies after a *Drosophila* C virus (DCV) infection. Viral load is increased in *Ect4* mutants resulting in higher mortality rates than wild-type. Overexpression of *Ect4* leads to a suppression of virus replication and thus improves the survival rate of the animals. Ect4 is required for the viral induction of STAT-responsive genes, *TotA* and *TotM*. Furthermore, Ect4 interacts with Stat92E, affecting the tyrosine phosphorylation and nuclear translocation of Stat92E in S2 cells. Altogether, our study identifies the adaptor protein Ect4 of the Toll pathway contributes to resistance to viral infection and regulates JAK/STAT signaling pathway.

## 1 Introduction

Viral infections seriously threaten human health and majorly cause mortality worldwide. The fruit fly *Drosophila melanogaster* has been proven to be a powerful model for deciphering antiviral immune responses (1). To defend against viruses, *Drosophila* relies on antiviral immunity, including RNA interference (RNAi) and inducible responses (2). Studies have shown RNAi to play a major role in defense against viruses in *Drosophila*. After detecting viral RNAs, Dicer-2 processes them into small interfering RNAs (siRNAs), which are loaded onto the RISC (RNA-induced silencing complex) complex that contains Argonaute-2 (AGO2) to target the complementary viral sequences for silencing (3). Two cellular processes, autophagy and phagocytosis are involved in antiviral defense. Autophagy plays a relatively minor role in antiviral defenses, whereas phagocytosis only contributes to virus-specific immune responses (4). Genetic studies suggest an involvement of the evolutionarily conserved innate immune pathways in controlling viral infections. The cytosolic DNA sensor cyclic GMP-AMP (cGAMP) synthase (CGAS) catalyzes 2’ 3’-cGAMP and activates Sting-dependent antiviral responses in mammals. Recently a class of cGAS-like receptors (cGLRs) was identified in *Drosophila* playing key roles in defense against viral infections (5, 6). Inactivation of the Toll pathway results in increased susceptibility to *Drosophila* X virus (DXV) infection, and the Imd pathway is required for an effective antiviral immune response against Cricket Paralysis Virus (CrPV) (7, 8). Another pathway contributing to *Drosophila* antiviral immunity involves Janus Kinase/Signal Transducer and Activator of Transcription (JAK/STAT) signaling (9). Deficiency in JAK/STAT pathway leads to increased DCV viral loads and higher mortality. In contrast to the Toll and Imd pathways, the JAK/STAT pathway is often activated by different types of stresses, such as mechanical pressure, heat shock, septic wounds, UV irradiation, and damage-associated molecular patterns (DAMPs) released from dead cells, instead of sensing microorganisms (10).

The evolutionarily conserved JAK/STAT pathway plays roles in various biological processes, including hematopoiesis, stress responses, and innate immunity (11–13). Dysregulation of the JAK/STAT pathway has been associated with several human diseases, such as autoimmune disease, allergy, and cancer (14–16). In *Drosophila*, JAK/STAT signaling is initiated by three cytokines of the Unpaired (Upd) family (Upd1, Upd2, and Upd3). The binding of Upd induces Domeless (Dome) dimerization and activation of the receptor-associated JAK molecules (termed Hopscotch). Activated Hopscotch then phosphorylates Dome, creating a docking site for the single *Drosophila* STAT family transcription factor, Stat92E. Phosphorylated Stat92E migrates into the nucleus in dimers, promoting target genes transcription (17). Infection with DCV has been shown to trigger the expression of JAK/STAT-dependent genes, including *virus-induced RNA 1* (*vir-1*) and stress response genes *Turandot A* and *M* (*TotA* and *TotM*) (18). Although the function of these JAK/STAT-dependent genes in *Drosophila* remains unknown, JAK/STAT signaling has been proposed to be involved in host resistance and tolerance to viral or parasitoid challenges (10, 19).

The Ectoderm-expressed 4 (Ect4) protein is evolutionarily conserved from arthropods to mammals (20). The mammalian Ect4 orthologue, Sterile-alpha and Armadillo motif-containing protein (SARM) has been identified as a negative regulator of TLR-mediated NF-κB activation and to mediate axonal death (21, 22). In *Drosophila* and Pacific white shrimp (*Litopenaeus vannamei*), the production of antimicrobial peptides (AMPs) was downregulated by Ect4 and LvSarm (23, 24), suggesting the involvement of Ect4 homologs in Toll pathway suppression is conserved in crustaceans and mammals. Interestingly, in invertebrate species including *C. elegans*, *Drosophila*, and *L. vannamei*, Ect4 homologs were demonstrated to play a positive role in host defense against pathogen infections (24–26). The positive and negative contributions to innate immunity suggested that the invertebrate Ect4 homologs are also involved in immune defense independent of the Toll pathway. This study investigated the role of Ect4 in antiviral defense against DCV infection. As a result, *Ect4* mutant flies exhibit increased susceptibility to infection by DCV, whereas overexpression of *Ect4* confers resistance against DCV infection; *Ect4* regulates the expression of JAK/STAT pathway target genes *TotA* and *TotM*; Ect4 interacts with Stat92E to alter the tyrosine phosphorylation status of Stat92E.

## 2 Materials and methods

### 2.1 Fly strains and mutant generation

*w^1118^* flies were used as wild-type control. The *w^IR^*; *dcr-2^L811fsX^* mutant flies have been previously described (27). *ubi-Gal4,tub-Gal80^ts^* was a gift from Dr. D. Ferrandon. *hop^Tum-l^*, *ppl-Gal4*, *da-Gal4*, *hs-Gal4* were obtained from Bloomington Stock center. The generation of transgenic *UAS-Ect4* and *U6:3-gRNA-Ect4* lines was performed as previously described (28). *Ect4-IR* was obtained from NIG-FLY stocks (HMJ30091). For the generation of Ect4 mutant lines, transgenic U*6:3-gRNA-Ect4* flies were crossed with the *nos-Cas9* flies to get male F_0_ (*nos-Cas9/+*; *U6:3-gRNA-Ect4/+*) that were crossed with *w^1118^*; *TM3*, *Sb/TM6B*, *Tb* to obtain F_1_ progenies. Singular F_1_ flies were crossed with *w^1118^*; *TM3*, *Sb/TM6B*, *Tb.* PCR products amplified from F_1_ flies before being cloned into the *pMD19-T* vector according to the manufacturer’s instructions (TAKARA) for mutation identification.

### 2.2 Plasmid construction

*pAC5.1-Ect4-Flag* was made by cloning Ect4 cDNA into *pAC5.1-Flag* vectors. For *pAC5.1-Ect4-GFP* constructs, the EGFP fragment was amplified from *pEGFP-C1* and assembled with the *Ect4* fragment into *pAC5.1-V5* vectors using ClonExpress MultiS One Step Cloning Kit (Vazyme). *Stat92E* cDNA was inserted in pAC5.1-HA to generate *pAC5.1-Stat92E-HA*. The hop (or *Dome*) cDNA was inserted in *pAC5.1-V5* to generate *pAC5.1-hop-V5* (or *pAC5.1-Dome-V5*). For the truncated *Ect4* constructs, ARM domain (aa 318-701), SAM domain (aa 680-826), and TIR domain (aa 829-1360) were amplified from *pAC5.1-Ect4-Flag* before assembled into *pAC5.1-Flag* empty vector, respectively.

### 2.3 Cell transfection, co-immuno-precipitation, and Western blot

S2 cells were cultured at 25°C in Sf-900™ III SFM (Gibco). All S2 cell transfection experiments were carried out with the Effectene Transfection Reagent (QIAGEN). For a co-immunoprecipitation assay, S2 cells were transfected with different plasmids. After 48h, cells were collected and lysed in lysis buffer (150 mM NaCl, 25mM Tris-HCL, pH 7.4, 5% glycerol, 1% NP-40, 1mM EDTA, complete protease inhibitor cocktail tablets [Roche] and phosphatase inhibitor cocktail tablets [Roche]). Lysates were incubated overnight at 4°C with Anti-Flag M2 affinity gel (Sigma) or EZview Red Anti-HA Affinity Gel (Sigma). After centrifugation, pellets were washed with 1ml lysis buffer three times before resuspension in 2X Laemmli SDS-PAGE buffer and detection by Western blot. Western blot was performed according to standard procedures.

Primary antibodies: Mouse anti-V5 (1:8000, Proteintech 66007-1-Ig); mouse anti-HA (1:8000, Milipore 05-904); mouse anti-α-Tubulin (1:20,000 Sigma T8203); goat anti-Stat (1:5000, Santa Cruz Biotechnology dN-17); mouse anti-FLAG (1:8000, Sigma F3165); rabbit anti-DCV (1:5000, Abcam ab92954); mouse anti-PY20 (1:2000, Abcam ab10321). Secondary antibodies: HRP-linked anti-mouse IgG (1:8000, Cell Signaling Technology 7076P2); HRP-linked anti-rabbit IgG (1:5000, Cell Signaling Technology 7074P2); HRP-linked anti-goat IgG (1:5000 Millipore AP106P); Alexa Fluor 555 goat anti-mouse IgG (1:500, life technologies A21422).

### 2.4 RNA analysis

According to the manufacturer’s instructions, total RNA was extracted from infected flies using RNAiso Plus (TAKARA), and cDNA was synthesized with the HiScript II Q RT SuperMix (Vazyme). The ChamQ SYBR qPCR Master Mix (Vazyme) was used for quantitative. Expression of the gene of interest was normalized to the Rpl32 RNA level. The following primers were used for qPCR: *RpL32* (forward 5’-GACGCTTCAAGGGACAGTATCTG-3’; reverse 5’-AAACGCGGTTCTGCATGAG-3’), *vir-1* (forward 5’-GATCCCAATTTTCCCATCAA-3’; reverse 5’-GATTACAGCTGGGTGCACAA-3’), DCV (forward 5’-TCATCGGTATGCACATTGCT-3’; reverse 5’-CGCATAACCATGCTCTTCTG-3’), *TotA* (forward 5’-CCCTGAGGAACGGGAGAGTA-3’; reverse 5’-CTTTCCAACGATCCTCGCCT-3’), *TotM* (forward 5’-ACCGGAACATCGACAGCC-3’; reverse 5’-CCAGAATCCGCCTTGTGC-3’), *Ect4* (forward 5’-GCCTCCAGTATTACGGT-3’; reverse 5’-ATGTTTCTCCTGACTGATGA-3’), *Vago* (forward 5’-TGCAACTCTGGGAGGATAGC-3’; reverse 5’-AATTGCCCTGCGTCAGTTT-3’).

### 2.5 Virus infection

Virus stocks were prepared as described previously (29). All fly lines confirmed the absence of Wolbachia by PCR and were cured whenever necessary. For infection, 3-6 d old flies were anesthetized with CO_2_ and injected with PBS (Gibco) or virus suspension intra-thoracically using the Nanoject II injector (Drummond). Infected flies were monitored daily for survival rate or frozen for RNA analysis at the indicated time points.

### 2.6 Cell immunofluorescence and eye-pigmentation measurement

S2 cells were transfected with *pAC5.1-Ect4-GFP* and pAC5.1-Stat92E-HA plasmids, and approximately 1×10^6^ cells were transferred to 24 well plates containing coverslips 48 h after transfection. Twelve h later, cells were washed in 0.5ml PBS and fixed with 4% formaldehyde in PBS for 15 min, then washed twice in 0.5 PBT (PBS containing 0.1% Tween-20) before blocking with 5% BSA in TBST for 1 h. Cells were then incubated with primary antibody (anti-HA 1:1000) overnight at 4°C before 2×5 min TBST washes. The secondary antibody was incubated for 4 h at room temperature. Nuclei were stained with PBS with 10 μg/ml DAPI for 5 min. Immunostaining samples were photographed with a Zeiss confocal microscope.

For eye pigment assay, the heads of 50 female flies (2-3 **d** old, raised at 25°C) of each indicated genotype were homogenized in methanol (1 **ml**, acidified with 0.1% HCl). After centrifugation, the supernatants were measured for absorbance at 480 nm.

### 2.7 RNAi knockdown in S2 cells and drug treatment

dsRNA targeting *Ect4* and *GFP* were synthesized according to standard protocol. S2 cells were treated with a culture medium containing 10 μg/ml dsRNA for 3 d. After dsRNA treatment, a solution containing 2 mM H_2_O_2_ and 1 mM sodium vanadate (final concentrations; Sigma) pre-incubated for 15 min was added to S2 cells to induce tyrosine phosphorylated Stat92E for 30 min. Cell lysates were prepared with the lysis buffer before immune precipitation with an anti-Stat92E antibody at 4°C and incubated with Pierce Protein A/G Plus Agarose (Thermo Scientific) beads. Co-immuno-precipitated proteins were detected with an anti-Stat92E antibody or anti-PY20 antibody.

### 2.8 Statistical analysis

Survival data were analyzed by the Kaplan-Meier method using GraphPad Prism. Quantification of immunoblots was performed with ImageJ 1.51p. Altered protein levels were presented as normalized fold change compared to the control value. Statistical analysis was performed using the Student’s *t*-test. Grey value analysis was performed by the ZEN 2012 (blue edition) system. *P*-values below 0.05 were considered significantly different.

## 3 Results

### 3.1 Reduced resistance of Ect4 mutants to DCV infection

**Figure 1:**
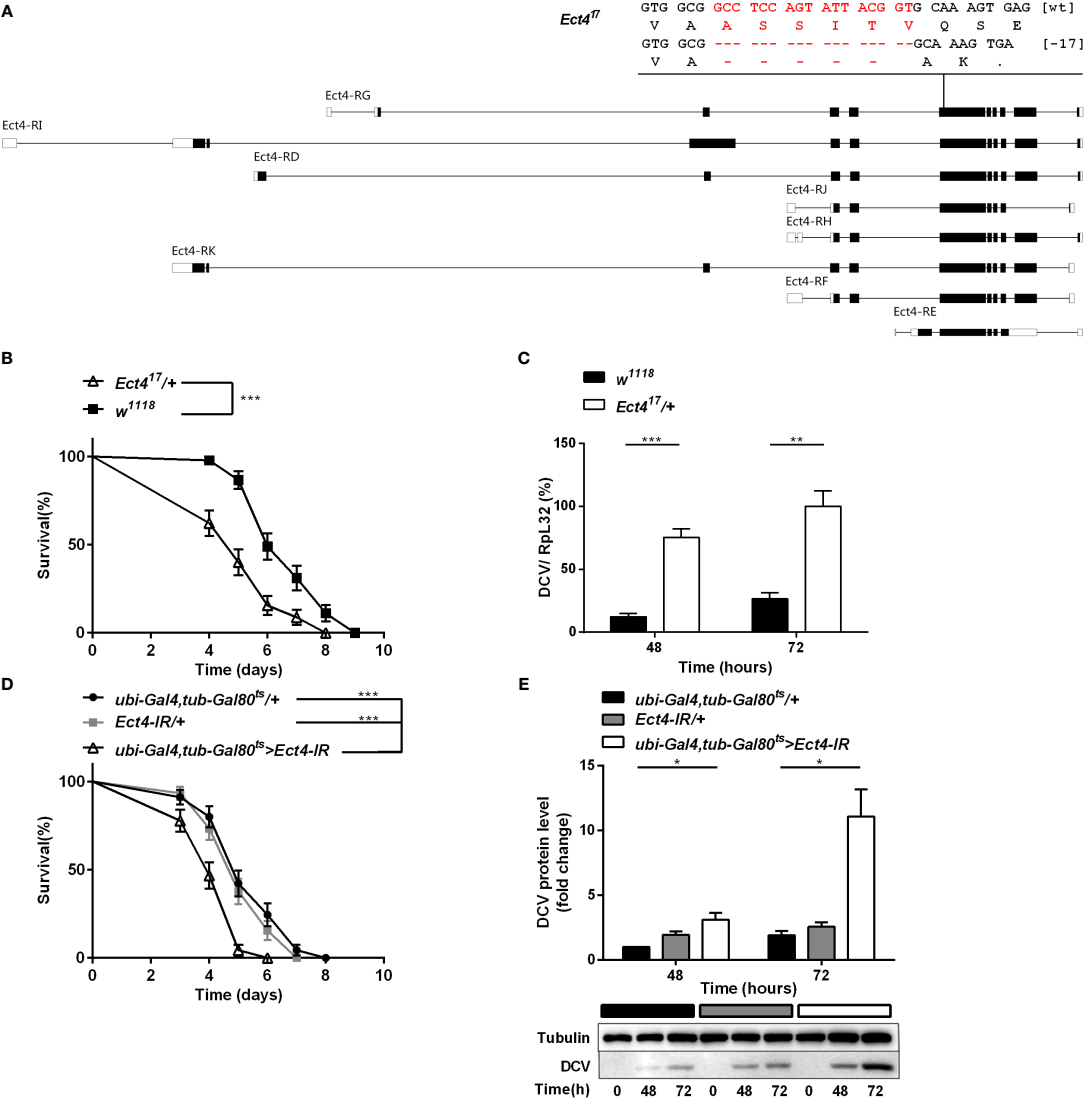
Depletion of *Ect4* in adult flies leads to a reduction in viral resistance upon DCV infection. **(A)** Schematic representation of deletions at exon of *Ect4* gene induced by CRISPR/Cas9. The deletion of 17 nucleotides (marked in red) caused a frameshift and created an early stop codons in the *Ect4^17^* mutant. Exons are represented by boxes, and introns by lines. UTRs are shown in white, and coding sequences are shown as black blocks. **(B)** Survival of *Ect4* mutants and wild-type flies was monitored daily at 25°C. **(C)** Quantitative RT-PCR analysis of the accumulation of viral RNA at 48 and 72 h post-infection in wild-type and *Ect4* mutant flies. **(D)** Survival of flies carrying the temperature-dependent *Ect4* knockdown system and genetic control flies upon DCV infection at 29°C. **(E)** Immunoblot of the accumulation of DCV capsid polyprotein in *Ect4*-RNAi or control flies, color blocks represented the genotype as indicated. Data represent the means ± standard errors of 3 independent pools of 15 male flies **(B, D)** or 10 male flies **(C, E)** for each genotype. Log-rank test **(B, D)** and *t-*test **(C, E)**: *P< 0.05, **P< 0.01, ***P< 0.001.

To investigate the role of *Ect4* in *Drosophila* antiviral defense, an *Ect4* mutant line was generated with the CRISPR/Cas9 system. The mutation, *Ect4^17^*, covers a genomic deletion of 17 bp in the coding region of *Ect4* (**Figure 1A**). *Ect4^17^* homozygous mutants are lethal at the second instar larval stage as judged by examining the development of both homo- and heterozygous animals distinguished by a GFP marker (**Figure S1A**), and heterozygous mutants were used for further experiments.

Variation in the pastrel gene is associated with natural resistance to DCV infection in *D. melanogaster*. The non-synonymous single nucleotide polymorphism (SNP) position 598, located in the last exon, has the strongest effect on DCV susceptibility (30). Sequencing of the pastrel locus revealed that all the strains of *D. melanogaster* tested contained the susceptible allele (data not shown), thus limiting the effect of discordance in the SNP profile between different fly lines to the difference in DCV resistance.

*Ect4* transcription in *Ect4^17^/+* heterozygotes was reduced by 45% compared with the wild-type flies (**Figure S1B**). Wild-type and Ect4 mutant files were challenged with DCV by intra-thoraxic injection. *Ect4* mutants were more sensitive to infection than wild-type flies, with a significantly different mean survival of 5 and 6 d for *Ect4^17^/+* and *w^1118^* male flies, respectively (**Figure 1B**). Notably, a significant increase in the DCV viral loading was observed in *Ect4^17^/+* flies at 48 and 72 h post-infection (**Figure 1C**), indicating that *Ect4* mutants are more sensitive to DCV infection.

To consolidate the DCV sensitivity phenotype observed with heterozygous *Ect4* individuals, the temperature-sensitive Gal80ts allele (31) was used to knockdown Ect4 expression in adult flies by shifting the culture temperature from 18-20°C to 29°C before and during the infection of DCV. RT-qPCR shows that *Ect4* expression decreased after the temperature shift to 29°C (**Figure S3A**). As expected, flies with knockdown of *Ect4* succumbed earlier to DCV infection than the control flies (**Figure 1D**). Consistently, the down-regulation of *Ect4* increased viral proteins (**Figure 1E**). Since DCV replicates mainly in fat bodies (32), we employed a fat body-specific driver, ppl-Gal4, to knock down Ect4 expression in the fat body. Specific depletion of *Ect4* in the fat body under the control of *ppl-Gal4* also affected the survival rate and viral load upon DCV infection (**Figures S2A, B**). The decreased survival rate was correlated with the increased viral burden in Ect4-RNAi flies.

### 3.2 Ect4 protects flies from DCV infection

**Figure 2:**
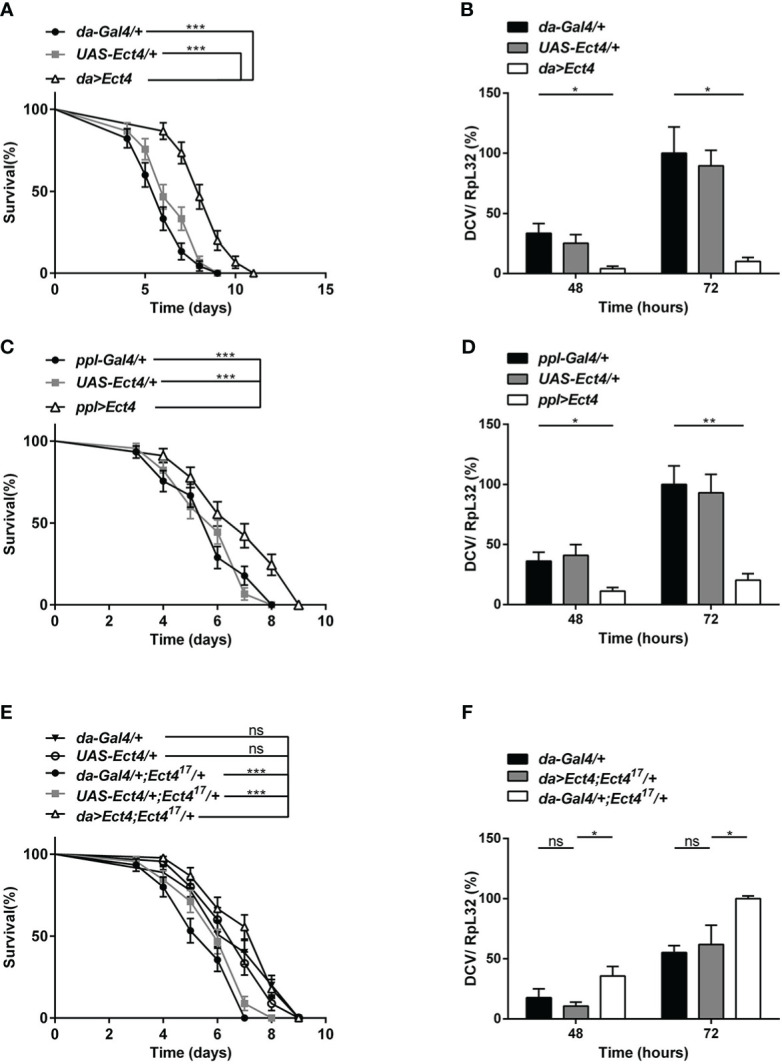
Overexpression of *Ect4* provides strong protection against DCV. **(A)** Survival of flies expressing *Ect4* transgene in whole flies by *da-Gal4* driver and control flies following DCV infection. **(B)** Quantitative RT-PCR analysis of the accumulation of viral RNA at 48 and 72 h post-infection in *Ect4* overexpression and control flies. **(C)** Survival of flies expressing *Ect4* transgene specifically in the fat body by the *ppl-Gal4* driver and control flies following DCV infection. **(D)** Quantitative RT-PCR analysis of the accumulation of viral RNA at 48 and 72 h post-infection in *Ect4* overexpression and control flies, specifically in the fat body. **(E)** Survival of *Ect4* mutant flies expressing *Ect4* transgene under the control of *da-Gal4* and control flies post-DCV infection. **(F)** Quantitative RT-PCR analysis of the accumulation of viral RNA at 48 and 72 h post-infection in control or *Ect4* mutant flies expressing *Ect4* transgene. Data represent the means ± standard errors of 3 independent pools of 15 male flies **(A, C, E)** or 10 male flies **(B, D, F)** for each genotype. Log-rank test **(A, C, E)** and *t-*test **(B, D, F)**: *P< 0.05, **P< 0.01, ***P< 0.001, ns, not significant.

To verify the specificity of the function of *Ect4* in DCV infection, *UAS-Ect4* transgenic flies were crossed with a ubiquitous *Gal4* driver, *da-Gal4*, to express the *Ect4* transgene ectopically. Remarkably, ubiquitous overexpression of *Ect4* promoted survival after the viral challenge (**Figure 2A**). Further, the increased dose of *Ect4* led to decreased viral burden in infected flies (**Figure 2B**). Interestingly, as shown in **Figures 2C, D**, flies overexpressing *Ect4*, specifically in the fat body using the *ppl-Gal4* driver, showed significantly more resistance to DCV infection than control flies and significantly decreased DCV replication levels. More importantly, rescue experiments by the expression of *Ect4* in *Ect4^17^/+* flies under the control of *da-Gal4* were performed to prove the specific role of *Ect4* in protecting flies from viral infections. The decreased survival rate of *Ect4* mutants after DCV infection, as well as increased viral load, was rescued to similar levels of the control flies following transgenic expression of *Ect4* in heterozygous *Ect4* mutants (**Figures 2E, F**). Similar results were obtained when a *hs-Gal4* driver was used for the rescue experiment. *Ect4* heterozygous mutant flies expressing *Ect4* under the control of *hs-Gal4* exhibited a decreased viral replication at 48 h post-infection. (**Figure S2C**). These results indicate that *Ect4* confers resistance against DCV infection and is required to control the accumulation of viruses.

### 3.3 Deficiency in Ect4 does not alter the activity of the siRNA pathway

**Figure 3:**
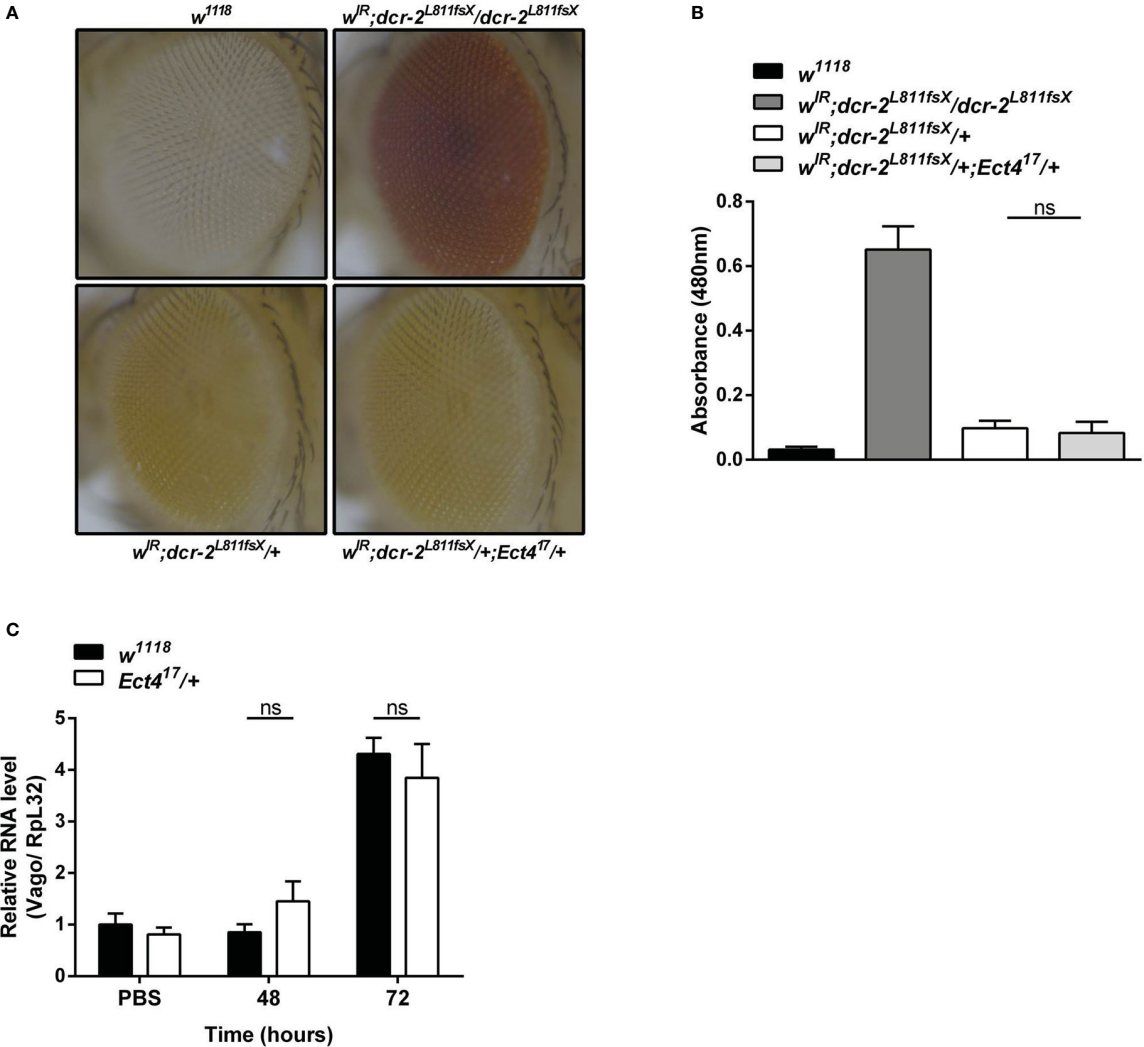
*Ect4* deficiency did not affect siRNA-mediated gene silencing. **(A)** Effects of altering dosages of *Ect4* in *w^IR^*; *dcr-2^L811fsX^* background. The eye color of a *white* null mutant fly (upper left panel) and a *white^+^* fly carrying *GMR>UAS-wIR* transgene and homozygous mutant for *dcr-2^L811fsX^* (upper right panel). The eye color of a fly carrying heterozygous *dcr-2^L811fsX^* mutation with (bottom right panel) or without (bottom left panel) one *Ect4* mutant allele. **(B)** Red-eye pigment levels of the indicated phenotype were determined by measuring absorbance at a wavelength of 480 nm (n = 50 for each group). **(C)** Expression levels of *Vago* at 48 and 72 h post-infection in wild-type or *Ect4* mutant flies challenged with DCV. Data represent the means ± standard errors of 3 independent pools of 50 female flies **(B)** or 10 male flies **(C)** for each genotype. The *t*-test **(B, C)**: ns, not significant.

RNA interference (RNAi) acts as the first line of defense against viruses in *Drosophila* (33). There was strong genetic evidence that one RNAi-related pathway, the siRNA pathway, plays a major role in antiviral immunity in *Drosophila* (7, 34). Since heterozygous *Ect4* mutant flies are hypersensitive to DCV infection, we asked whether the down-regulation of *Ect4* affects the function of the siRNA pathway. To address this question, siRNA pathway activity was monitored using an *in vivo* sensor assay, wherein the endogenous *white* gene is silenced by the expression of a hairpin dsRNA corresponding to an exon of *white*. Expression of *UAS-wIR* using the eye-specific driver *GMR-Gal4* alters eye pigmentation to a white color or pale orange if the silencing is incomplete. Studies have shown that the siRNA pathway is inactivated without *Dicer-2* (*Dcr-2*). Therefore eye pigmentation of a *Dcr-2* null mutant (*dcr-2^L811fsX^/dcr-2^L811fsX^*) in *GMR>UAS-wIR* background is red, whereas *Dcr-2* heterozygous mutants (*dcr-2^L811fsX/+^*) display pale orange (27). Our results show that mutation in *Ect4* did not lead to any changes in the eye pigmentation in *w^IR^; dcr-2^L811fsX^/+* (**Figures 3A, B**). Moreover, the expression of *Vago*, induced in DCV infection dependent on *Dicer-2* (33), did not differ between wild-type and *Ect4* mutant flies at 48 and 72 hpi (**Figure 3C**). These results suggest that *Ect4* does not directly affect the antiviral siRNA pathway.

### 3.4 Ect4 regulates the expression of JAK/STAT-dependent genes, *TotA*, and *TotM*

**Figure 4:**
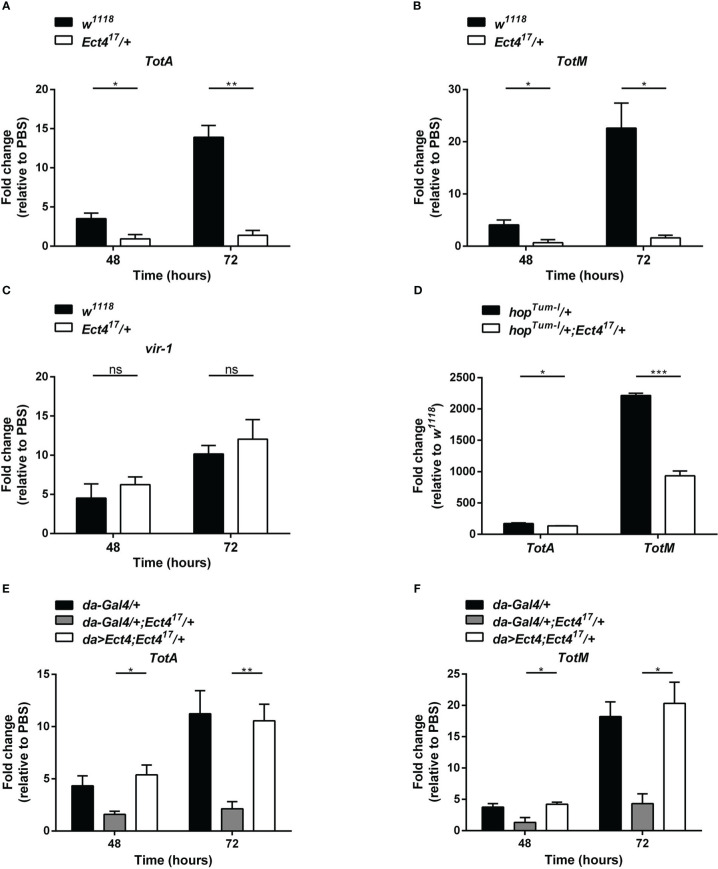
*Ect4* is required for *TotA* and *TotM* induction in response to DCV infection. **(A–C)** Expression of JAK/STAT-dependent gene *TotA*, *TotM*, and *vir-1* at 48 and 72 h after DCV infection determined by RT-qPCR in whole flies. Expression of the gene of interest was normalized to transcript levels of the housekeeping gene *Rpl32* and expressed as fold change relative to mock infection (PBS). **(D)** Expression levels of *TotA* and *TotM* on 3-5 d-old unchallenged flies carrying one copy of *hop^Tum-l^* allele with or without the *Ect4* mutant allele. Expression of *TotA* and *TotM* was normalized to transcript levels of the housekeeping gene *Rpl32* and expressed as fold change relative to wild-type (*w^1118^*) flies. **(E, F)** Expression levels of *TotA* and *TotM* at 48 and 72 h in control flies or *Ect4* mutant flies overexpressing *Ect4* transgene under a ubiquitous *da-Gal4* driver upon DCV infection. Data represent the means ± standard errors of 3 independent pools of 10 male flies **(A–F)** for each genotype. *T-*test **(A–F)**: *P< 0.05, **P< 0.01, ***P< 0.001, ns, not significant.

The JAK/STAT pathway was shown to contribute to the antiviral response in *Drosophila* (9), where several genes are induced following viral infection *via* the JAK/STAT pathway including *virus-induced RNA-1* (*vir-1*), the stress-induced genes *Turandot A* and *M* (*TotA* and *TotM*) (18). To examine whether the downregulation of *Ect4* affected JAK/STAT pathway activation, we examined the expression of *vir-1*, *TotA*, and *TotM* by RT-qPCR at 48 and 72 h after DCV infection (hpi). As previously reported, DCV infection induced a strong up-regulation of *vir-1*, *TotA*, and *TotM* in wild-type flies (18). However, *vir-1* induction in response to DCV infection in *Ect4* mutant flies was indistinguishable from the control (**Figure 4C**). Similar results were observed using a ubiquitous temperature-sensitive Gal4 driver, *ubi-Gal4* (**Figures S3B, C**). A genetic interaction experiment was performed to assess further the relationship between Ect4 and the JAK/STAT pathway. *TotA* and *TotM* were expressed in flies carrying a JAK gain-of-function allele *Tum-l* (*hop^Tum-l^*), which encodes a hyperactive JAK kinase due to a G341E substitution (35). Reducing the dosage of *Ect4* by half resulted in a large reduction of the RNA levels of *TotA* and *TotM* in *hop^Tum-l^* flies (**Figure 4D**). The *TotA* and *TotM* response was also attenuated in the fat body of flies where *Ect4* was downregulated by expressing the *Ect4-IR* transgene using a *ppl-Gal4* driver (**Figures S3D, E**). This TotA and TotM expression reduction was rescued by ubiquitously expressed *Ect4* (**Figures 4E, F**). Together, these results suggest that *Ect4* genetically interacts with the JAK/STAT pathway to regulate the expression of *TotA* and *TotM* in response to DCV infection.

### 3.5 Ect4 physically interacts with Stat92E

**Figure 5:**
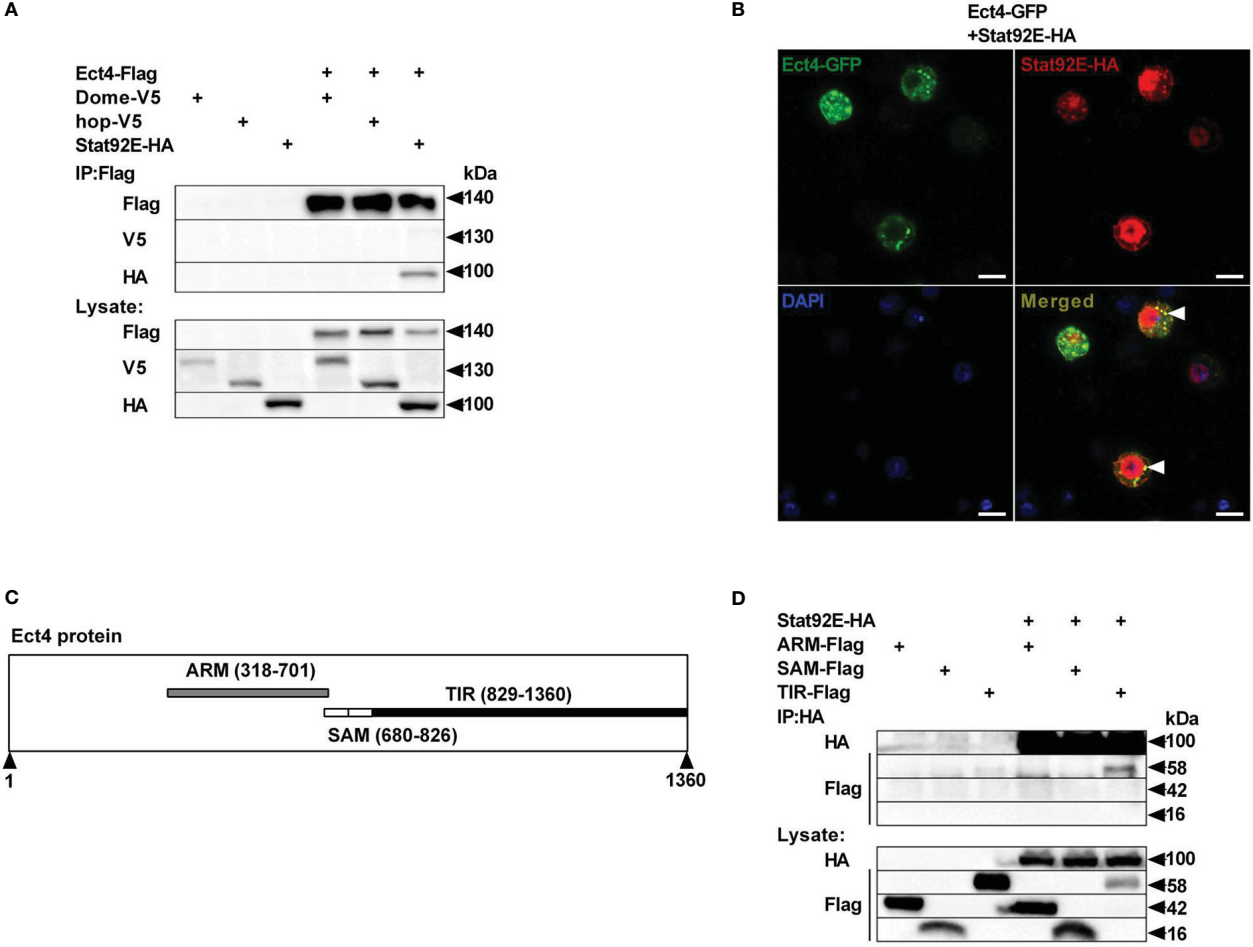
Ect4 co-localizes and physically associates with Stat92E. **(A)** S2 cells were transfected with combinations of expression plasmids as indicated. Cell lysates were immunoprecipitated with anti-Flag beads, followed by immunoblot analysis with the indicated antibodies. **(B)** S2 cells were transfected with Ect4-GFP in combination with Stat92E-HA, stained with DAPI (blue) and anti-HA antibody (Red), and imaged by confocal microscopy. Ect4 was co-localized with Stat92E in the cytoplasm (white arrow). **(C)** Ect4 protein includes ARM (gray), SAM (white), and TIR (black) domain. **(D)** S2 cells were cotransfected with Stat92E-HA and domains of Ect4. Cell lysates were immunoprecipitated with anti-HA beads and immunoblot analysis with the indicated antibodies. Scale bar: 10 μm.

To unravel the molecular mechanism underlying the relationship between *Ect4* and JAK/STAT pathway, we examined whether Ect4 interacted with any known components of the JAK/STAT pathway. Differentially tagged forms of JAK/STAT pathway components and Ect4 were expressed in S2 cells, and co-immunoprecipitation studies were performed. As shown in **Figure 5A**, Ect4 is associated with the transcription factor Stat92E but not other key components (Hop or Dome) of the JAK/STAT pathway. Consistent with previous findings (36), Stat92E protein was located both in the cytoplasm and nucleus as visualized by immunofluorescence staining. Since the green fluorescent protein (GFP)-Ect4 fusion protein was localized in the cytoplasm, the interaction between the two proteins occurs in the cytoplasm (**Figure 5B**).

Ect4 protein harbor three different domains: ARM (Armadillo motif) domains followed by two SAM (Sterile Alpha motif) domains and TIR (Toll -Interleukin-1 receptor) domain (**Figure 5C**). To further investigate the molecular basis of the interaction between Ect4 and Stat92E, a series of truncated forms of Ect4 were generated. Co-immuno-precipitation studies showed that ARM and SAM domains were likely not required for Ect4 to interact with Stat92E, whereas the TIR domain was essential since only the TIR domain co-immuno-precipitated with Stat92E (**Figure 5D**). Together, these results suggest that Ect4 may regulate the JAK/STAT signaling activity by interacting with Stat92E.

### 3.6 Ect4 is required for phosphorylation and nuclear translocation of Stat92E

**Figure 6:**
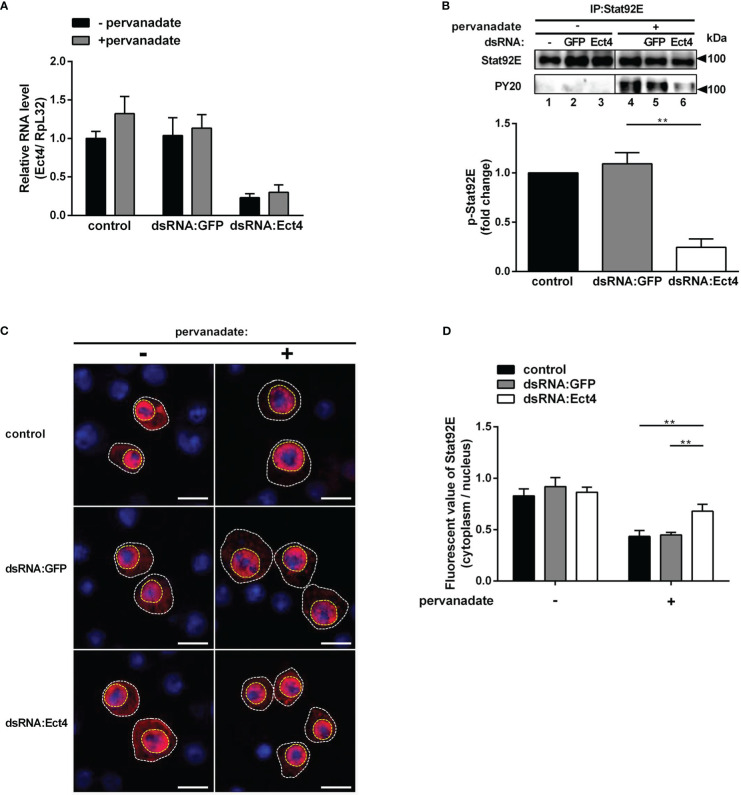
Knockdown of *Ect4* mRNA decreased the phosphorylation of Stat92E. **(A)** S2 cells were pretreated with or without dsRNA targeting GFP or Ect4 transcripts for 72 h, and measurement of *Ect4* mRNA by RT-qPCR to confirm RNAi efficiency. **(B)** After dsRNA treatment, S2 cells were either unstimulated or treated with pervanadate. Stat92E proteins were immunoprecipitated with Stat92E antibody, followed by immunoblot analysis with the Stat92E or PY20 antibodies. In the absence of pervanadate, phosphorylated-tyrosine Stat92E (p-Stat92E) was undetectable (lane 1-3). In contrast, after pervanadate treatment, p-Stat92E increased to levels detectable by western blot (lanes 4-6). The phosphorylated Stat92E was quantified from n = 3 independent experiments. **(C)** After transfection with Stat92E-HA, S2 cells were treated with or without dsRNA targeting GFP or Ect4 for 72 h and then left unstimulated or treated with pervanadate. Cells were stained with anti-HA antibody (red) and DAPI (blue) and imaged by confocal microscopy. **(D)** Quantification assays of the ratio between the fluorescent signal intensity of Stat92E in the nucleus (surrounded by a yellow dashed line) and in the cytoplasm (the area in the white dotted line subtracts the area from the yellow dashed line). Cell samples collected from **(C)**, n=15. Scale bar: 10 μm. Data represent the means ± standard errors. The *t-*test **(B, D)**: **P< 0.01.

As described thus far, we show that *Ect4* regulates the expression of JAK/STAT-dependent genes *TotA* and *TotM* and is associated with Stat92E. It is intriguing to predict that Ect4 may affect Stat92E phosphorylation. To test this hypothesis, we employed an RNAi approach to knock down *Ect4* in S2 cells (**Figure 6A**). Previous studies have shown that tyrosine residues of Stat92E are phosphorylated after treatment of S2 cells with pervanadate, which activates Stat92E in a ligand-independent manner, while activation is not present in untreated cells (36, 37). As shown in **Figure 6**, treatment with dsRNA targeting *Ect4* mRNA resulted in a significant reduction of tyrosine phosphorylated Stat92E upon pervanadate treatment, as compared with the control using dsRNA targeting GFP. It was noted that upon DCV infection of S2 cells, phosphorylated Stat92E (p-Stat92E) was not detected by immunostaining, likely due to the transient activity of p-Stat92E dimers. Therefore, is Ect4 required for the nuclear translocation of Stat92E? As expected, reduced nuclear translocation of Stat92E in response to the pervanadate stimulus was detected in cells treated with *Ect4* RNAi (**Figures 6C, D**).

## 4 Discussion

The innate immune system processes pathogen-induced pathways that detect the pathogen and induce the expression of antiviral effectors that control its proliferation (38). Consequently, it is expected that insufficient resistance mechanisms will lead to an increase in viral load, increased morbidity, and reduced survival. An in-depth understanding of antiviral resistance is important for developing novel methods for treating viral infections and other diseases. Nevertheless, mechanisms of resistance still need to be clearly understood.

Invertebrate *Ect4* orthologues play a positive role in innate immunity. The Ect4 orthologue in *C. elegans* (*TIR-1*) and *L. vannamei* (*LvSarm*) were required to express antimicrobial peptides. Depletion of both led to decreased survival of the animals upon bacterial infections (24, 26, 39). Kemp et al. (40) reported that Ect4 is responsive to DCV infection. And about a 2-fold increase of Ect4 transcription at 72 hpi was observed in this study (data not shown), indicating that Ect4 participated in the immune response upon DCV infection. Furthermore, our study showed that Ect4 contributes to resistance and regulates JAK/STAT signaling in *Drosophila.* Flies with down-regulated *Ect4* showed significantly elevated viral replication and earlier mortality after the DCV challenge, and a high level of *Ect4* expression was associated with increased resistance to DCV.

Compared with invertebrate Ect4 orthologues, the mammalian orthologue SARM acts as a negative regulator of TLR signaling and is not directly antiviral, as mice lacking SARM show enhanced survival after Bunyavirus infection (41) because *SARM* family members have acquired diverse biological functions during evolution. For example, while *Ect4* is essential for development in *Drosophila*, *SARM1* is redundant for viability in mice (21). A previous study revealed that SARM is expressed mainly in the mouse brain, whereas its expression in other tissues, such as the spleen and the lymph node, was low (42). However, due to the lack of suitable anti-Ect4 antibodies, detecting Ect4 protein expression in *Drosophila* tissue sections was unsuccessful in the present study. Our results show that ectopic overexpression or knockdown of Ect4 in the fat body has a positive or negative effect on immune resistance upon DCV infection, suggesting that *Ect4* may regulate antiviral immune system function mainly in the fat body.

Our study demonstrated that down-regulation in Ect4 does not directly interfere with the siRNA pathway. Instead, Ect4 regulates JAK/STAT dependent gene expression, *TotA*, and *TotM*, in response to DCV infection. A previous study revealed that the proper level of JAK/STAT signaling activation is required for normal immune response: hyper-activation of JAK/STAT triggered early mortality and loss of function mutations of *hop* in flies causing reduced JAK/STAT activation in flies, also decreasing resistance upon a challenge with DCV (18, 43). Despite being elicited by DCV and commonly used as a read-out of JAK/STAT activation, the function of *TotA* and *TotM* in *Drosophila* remains unclear. The protein products encoded by the Turandot gene family are protein chaperones or signaling molecules, which are produced in the fat body and secreted into the hemolymph (44, 45). This inflammatory response is reminiscent of the acute phase response in mammals, which can be activated by infection and produce acute phase protein. These proteins are involved in the immune responses, including host defense, vascular permeability, and coagulation.

Furthermore, TotM enhanced tolerance against fungal sexually transmitted infections (STIs), and *TotA* confers resistance to heat stress (45, 46). The *Tot* gene family regulates diverse fly physiology aspects that coordinate resistance or tolerance to immune challenges. Indeed, the present study showed that *Ect4* is required for virus-induced expression of *TotA* and *TotM* genes. However, *Ect4* is dispensable for *vir-1* induction in response to DCV infection. It is likely that different factors are involved in the regulation of JAK/STAT downstream genes and that less p-Stat92E in *Ect4* RNAi flies sufficient for inducing *vir-1* expression, which requires a lower threshold of STAT activity.

Ect4 interacts with Stat92E in S2 cells through the highly conserved TIR domain of SARM family origin, which have roles in cell death and neuronal destruction in mammals (47). As an adaptor, SARM has been reported that interact with the mitochondrial antiviral-signaling protein MAVS in the mitochondria to mediate cell death during virus infection (41). Mitochondrial localization of tyrosine-phosphorylated STAT5, a homolog of Stat92E in mammalian, has been supposed to modulate cellular metabolism in cytokine-stimulated cells (48, 49). We show that down-regulation of *Ect4* reduced phosphorylated Stat92E upon pervanadate treatment in S2 cells, which suggests a role of Ect4 in regulating cell death through the modulation of JAK/STAT *via* the interaction with Stat92E.

In addition to antiviral immune defense, apoptosis is a conserved mechanism of programmed cell death that can prevent the infection before viral replication is completed (50, 51). Our *in vivo* study revealed that Ect4 mutants showed enhanced mortality and increased viral load upon DCV challenge. We seek to further elucidate the unknown mechanisms of antiviral response in *Drosophila* by assessing whether *Ect4* affects host resistance to viral infection by regulating cell death.

Our results demonstrate the novel roles for *Drosophila* Ect4 in regulation of JAK/STAT signaling pathway and protection against DCV infection. It is still unclear if Ect4 also participated in the control of other virus infection. The contribution of JAK/STAT signaling to *Drosophila* antiviral protection is virus-specific. Although JAK/STAT pathway can be activated by RNA viruses, including DCV, CrPV, FHV, and DXV, it is only required for resistance against two *Dicistroviridae* family members, DCV and CrPV (18). Our data suggest that Ect4 is required for phosphorylation and nuclear translocation of Stat92E. Future studies should investigate if the involvement of *Ect4* in activating the JAK/STAT pathway impart resistance in *Drosophila* to other virus infection.

The tight regulation of immune-related signal transduction cascades is essential for the defense against a wide range of pathogens. However, although the key components of the JAK/STAT pathway have been identified, the ‘non-core’ pathway activity regulators are less known. In mammals, poly (ADP-ribose) polymerase PARP9 was recently reported as a noncanonical sensor for RNA viruses that depends on the PI3K/AKT3 pathway to produce antiviral type I interferon (52). PARP9 interacted with the E3 ubiquitin ligase DTX3L and STAT1 functioned as a chaperone to enhance levels of the PARP9-DTX3L protein complex and STAT1-mediated interferon-stimulated gene expression (53). Another E3 ubiquitin ligase TRIM18 recruited protein phosphatase 1A (PPM1A), a negative regulator of STAT1, to dampen type I interferon-mediated antiviral innate immunity for promoting virus infection (54, 55). Given the conserved nature of the JAK/STAT pathway, *Drosophila* homologs of PARP9 and TRIM18 are potential candidates for JAK/STAT pathway regulators. It will be intriguing to investigate whether other factors or pathways are involved in *Ect4*-mediated JAK/STAT pathway modulation and defense against viral infection. Further exploration will yield more insights into the current understanding of the JAK/STAT pathway immune regulatory mechanism and contributes to establishing an immune signaling network.

## Data availability statement

The raw data supporting the conclusions of this article will be made available by the authors, without undue reservation.

## Author contributions

ZH and PX carried out experiments. SG contributed to data collection. YH analyzed confocal images. YL, SY, and FS conducted statistical analysis. KA and W-MD revised the manuscript. JL and RJ contributed to the conception and design of the experiments. WW wrote the first draft of the manuscript. JC directed the studies. All authors contributed to the article and approved the submitted version.
