# Supplementary material for: Drosophila Ectoderm-expressed 4 modulates JAK/STAT pathway and protects flies against Drosophila C virus infection
Source: Front Immunol. 2023 Feb 3;14:1135625. doi: 10.3389/fimmu.2023.1135625 (PMC9937023; doi:10.3389/fimmu.2023.1135625)
Supplement: Supplementary file 1 [file DataSheet_1.docx]

**Supplemental materials:**


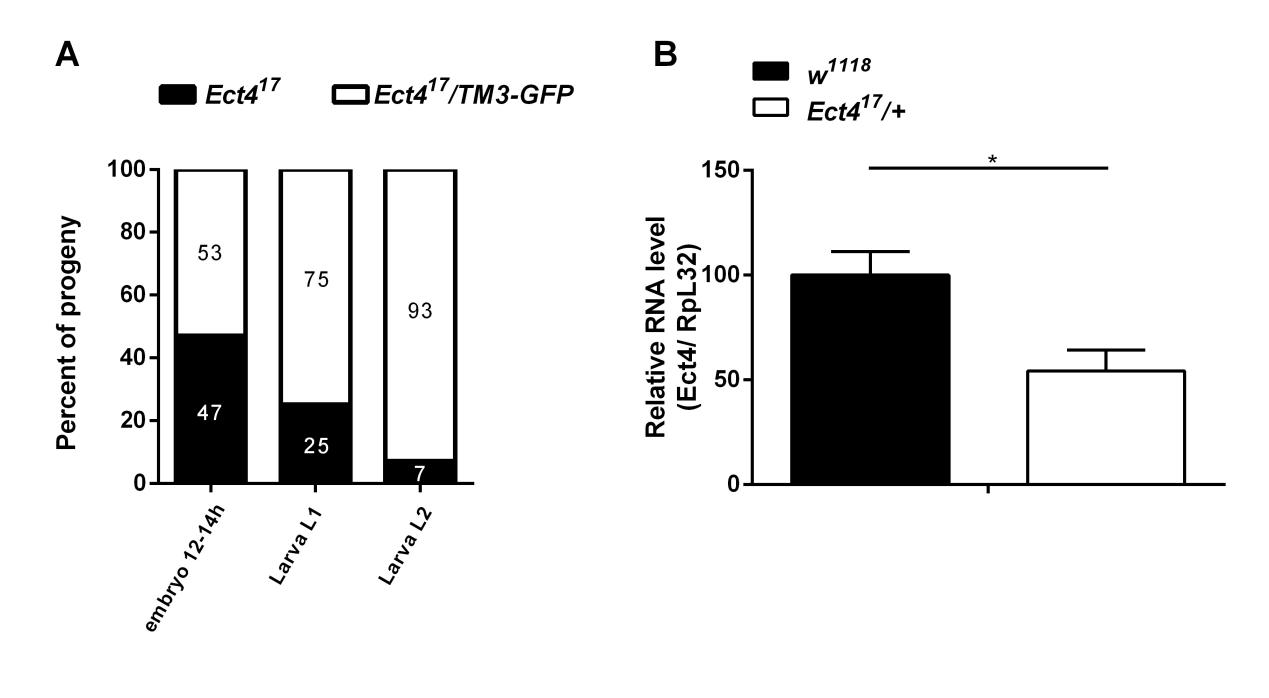


**Figure S1**. *Ect4* is required for viability during developments. (A) The ratio between *Ect4* mutant homozygous and heterozygous progeny was followed throughout development at 25℃ (n=205). (B) Expression levels of *Ect4* on 3 to 5-d-old *Ect4* heterozygous mutant (*Ect4^17^/+*) or wild-type flies (*w^1118^*). Data represent the means ± standard errors of 3 independent pools of 10 male flies. *t* test : **P*< 0.05.


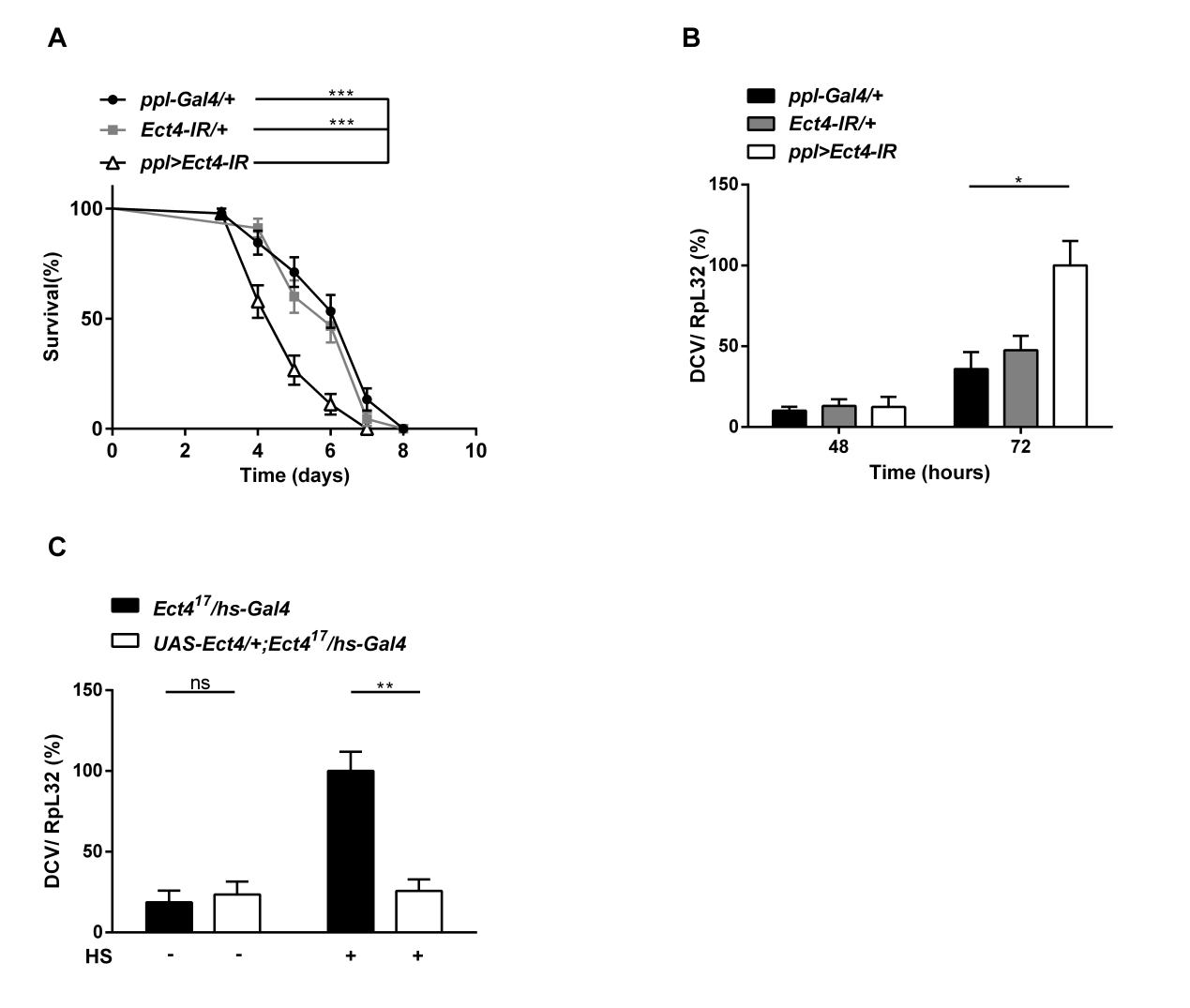


**Figure S2**. Knockdown of *Ect4,* specifically in the fat body, altered resistance upon DCV infection. (A) Survival of flies containing knockdown of *Ect4,* specifically in the fat body by the *ppl-Gal4* driver and genetic control flies upon DCV infection. (B) Quantitative RT-PCR analysis of the accumulation of viral RNA at 48 and 72 h post-infection in flies containing knockdown of *Ect4,* specifically in the fat body and genetic control flies. (C) Quantitative RT-PCR analysis of the accumulation of viral RNA at 48 h in control flies or flies overexpressing an *Ect4* transgene in *Ect4* mutant background. *Ect4* is expressed under the control of *hs-Gal4*. *UAS-Ect4/+*; *Ect4^17^/hs-Gal4* flies express *Ect4* after heat-shock (HS) treatment. Data represent the means ± standard errors of 3 independent pools of 15 male flies (A) or 10 male flies (B, C) for each genotype. Log-rank test (A) and *t-*test (B, C): **P*< 0.05, ***P*< 0.01, ****P*< 0.001, ns, not significant.





**Figure S3**. Knockdown of *Ect4* in flies suppressed *TotA* and *TotM* induction by DCV infection. (A) Quantitative RT-PCR analysis of the RNA level of *Ect4* to confirm RNAi efficiency. (B, C) Expression levels of *TotA* and *TotM* at 48 and 72 h in control flies or *Ect4* RNAi flies under the control of the Gal4-Gal80ts system upon DCV infection. (D, E) Expression levels of *TotA* and *TotM* at 48 and 72 h post-infection in flies containing knockdown of *Ect4,* specifically in the fat body and genetic control flies. Data represent the means ± standard errors of 3 independent pools of 10 male flies. The *t-*test: **P*< 0.05, ***P*< 0.01.
